# Association between electromyographical findings and intensive care unit mortality among mechanically ventilated acute respiratory distress syndrome patients under profound sedation

**DOI:** 10.5935/0103-507X.20190087

**Published:** 2019

**Authors:** Cassiano Teixeira, Régis Goulart Rosa, Juçara Gasparetto Maccari, Augusto Savi, Francisco Telechea Rotta

**Affiliations:** 1 Departamento de Clínica Médica e Reabilitação, Universidade Federal de Ciências da Saúde de Porto Alegre - Porto Alegre (RS), Brasil.; 2 Unidade de Terapia Intensiva, Hospital Moinhos de Vento - Porto Alegre (RS), Brasil.; 3 Departamento de Neurologia, Hospital Moinhos de Vento - Porto Alegre (RS), Brasil.; 4 Unidade de Terapia Intensiva, Hospital São Lucas, Pontifícia Universidade Católica do Rio Grande do Sul - Porto Alegre (RS), Brasil.

**Keywords:** Critical illness, Polyneuropathies, Prognosis, Mortality, Electromyography, Respiratory distress syndrome, Respiration, artificial, Sedation, Intensive care units

## Abstract

**Objective:**

To evaluate whether electromyographical findings could predict intensive care unit mortality among mechanically ventilated septic patients under profound sedation.

**Methods:**

A prospective cohort study that consecutively enrolled moderate-severe acute respiratory distress syndrome (partial pressure of oxygen/fraction of inspired oxygen < 200) patients who were ≥ 18 years of age, dependent on mechanical ventilation for ≥ 7 days, and under profound sedation (Richmond Agitation Sedation Scale ≤ -4) was conducted. Electromyographic studies of the limbs were performed in all patients between the 7^th^ and the 10^th^ day of mechanical ventilation. Sensory nerve action potentials were recorded from the median and sural nerves. The compound muscle action potentials were recorded from the median (abductor pollicis brevis muscle) and common peroneal (extensor digitorum brevis muscle) nerves.

**Results:**

Seventeen patients were enrolled during the seven months of the study. Nine patients (53%) had electromyographic signs of critical illness myopathy or neuropathy. The risk of death during the intensive care unit stay was increased in patients with electromyographical signs of critical illness myopathy or neuropathy in comparison to those without these diagnostics (77.7% *versus* 12.5%, log-rank p = 0.02).

**Conclusion:**

Electromyographical signs of critical illness myopathy or neuropathy between the 7^th^ and the 10^th^ day of mechanical ventilation may be associated with intensive care unit mortality among moderate-severe acute respiratory distress syndrome patients under profound sedation, in whom clinical strength assessment is not possible.

## INTRODUCTION

Critical illness myopathy (CIM) and critical illness neuropathy (CIP) are the main causes of neuromuscular weakness in the intensive care unit (ICU), and both are associated with sepsis.^(1,2)^ It is estimated that approximately 50% of patients with sepsis or acute respiratory distress syndrome (ARDS) develop ICU-acquired weakness (ICUAW),^(3)^ which is associated with prolonged ICU and hospital stays,^(4)^ prolonged duration of mechanical ventilation (MV),^(5)^ increased ICU and hospital mortality and healthcare-related hospitalization costs,^(6-8)^ and increased post-ICU discharge mortality.^(9,10)^

The diagnosis of ICUAW is primarily based on a clinical strength assessment, using tools such as the Medical Research Council scale.^(11)^ However, the clinical-based diagnosis requires patients to be awake and cooperative, preventing the assessment of 22 - 33% of patients.^(4,6,12)^ Conversely, the diagnosis of CIM and CIP is made by electrophysiological studies, and therefore, it is relatively independent of patient consciousness level and cooperation.^(11)^ Electromyographical screening, including the quantification of compound muscle action potentials (CMAP) and sensory nerve action potentials (SNAP), could serve as an alternative diagnostic tool for neuromuscular dysfunction among septic patients who are profoundly sedated or have other limitations impacting clinical assessment.^(11,13)^ Unfortunately, few studies have assessed the association between the electrophysiological findings of CIM and CIP and mortality in this specific subset of individuals.

Accordingly, the objective of this study was to evaluate whether electromyographical findings of CIM or CIP were able to predict ICU mortality among moderate-severe ARDS patients under profound sedation.

## METHODS

This prospective cohort study was performed during a seven-month period in the medical-surgical ICU of the *Complexo Hospitalar Santa Casa de Misericórdia*, Porto Alegre, Brazil. The local ethics committee approved the study, and written informed consent was obtained from proxies.

Patients ≥18 years of age requiring profound sedation (Richmond Agitation Sedation Scale - RASS ≤ -4) due to moderate-severe ARDS (*partial pressure* of *oxygen* /*fraction* of *inspired oxygen* - PaO_2_/FiO_2_ < 200)^(14)^ secondary to sepsis and under MV ≥ 7 days were consecutively included.

Exclusion criteria were: previous neuromuscular disorders, morbid obesity (body mass index greater than 40kg/m^2^), diabetes mellitus or cancer, lower limb disorders precluding nerve conduction study and electromyography (e.g., fractures, amputation, and plaster casts), brain death, RASS > -4, ICU admission due to neurologic conditions, and proxy refusal.

Electromyographic studies of the limbs were performed in all patients between the 7^th^ and the 10^th^ day of MV. The complete electromyographic tests performed on the patients consisted of conventional motor (median and common peroneal nerves) and sensory nerve (median and sural nerves) conduction studies. SNAPs were recorded from the median and sural nerves. Median sensory nerve conduction was recorded antidromically with ring electrodes on the proximal (-) and distal (+) interphalangeal joints of the third digit and stimulation on the volar surface of the wrist, 2 to 3cm proximal to the distal crease. For the sural nerve, the surface recording electrodes were placed immediately posteroinferior to the lateral malleolus (-) and 2 to 3cm distally along the lateral dorsum of the foot (+); the nerve was stimulated antidromically along the posterior surface of the leg (calf), slightly lateral to the midline and approximately 10 to 12cm from the active electrode (-). Compound muscle action potentials from abductor pollicis brevis and extensor digitorum brevis were recorded from electrodes placed over the muscle belly (-) and tendon (+), with simulation of the median nerve at the wrist on the volar surface, 2 to 3cm proximal to the distal crease, and at the elbow over the brachial pulse with the cathode at the volar crease, and of the common peroneal nerve at the ankle, 7 to 8cm from the recording electrodes, and below the head of the fibula (below the knee). Incremental electrical stimulation of the nerves was used until the best SNAP or CMAP amplitudes were obtained. If the clinical history and physical examination suggested a median nerve entrapment at the wrist or the median sensory nerve conduction study was abnormal, the median nerve was substituted with the ulnar nerve. Electromyography was recorded from coaxial needle electrodes in the tibialis anterior, quadriceps femori, abductor pollicis brevis, and deltoid muscles; additional muscles were studied in some patients.

Surface temperature was kept above 33ºC for the nerve conduction studies, with the aid of heat packs if necessary. Nerve conduction studies were considered abnormal if the CMAP or SNAP amplitude of at least two nerves of two limbs was reduced below two standard deviations of the lower limit of normal.

Critical illness neuropathy and CIM were distinguished by the following features: nerve conduction (reduced SNAP amplitude in CIP, and normal SNAP amplitude in CIM), needle electromyography (CIP: large and polyphasic motor unit potentials, and reduced recruitment; CIM: small polyphasic motor unit potentials, and early recruitment), and direct muscle stimulation (normal in CIP, and absent or reduced in CIM).

Patient treatment, including electrolyte (sodium, potassium, magnesium, and phosphorus) correction and blood glucose control, was performed according to accepted standards. Intravenous insulin, preferably using a pump, was started if the blood glucose level exceeded 200mg/dL, with a target of less than 160mg/dL.^(15)^ Intensive care unit staff members were in charge of hemodynamic, ventilatory and dialysis management.

Intensivists and clinical neurophysiologists were unaware of each other's diagnoses. All electromyographical recordings performed by the neurophysiologist hospital team were re-examined by one expert physician for quality assessment.

Data collected included age, sex, previous diseases, cause of admission, Acute Physiologic and Chronic Health Evaluation (APACHE)-II, and all-cause mortality. Information on the use of vasopressors (norepinephrine) and the need for dialysis and the Sequential Organ Failure Assessment (SOFA) at day 7 of MV were also collected.

We expressed continuous variables as the median ± standard deviation or percentage. We assessed the mortality effect using Kaplan-Meier curves and the log-rank test. A p value of < 0.05 was used to define a statistically significant difference. The statistical analysis was conducted with STATA version 12 (StataCorp LP, College Station, TX, USA).

## RESULTS

**Figure 1:**
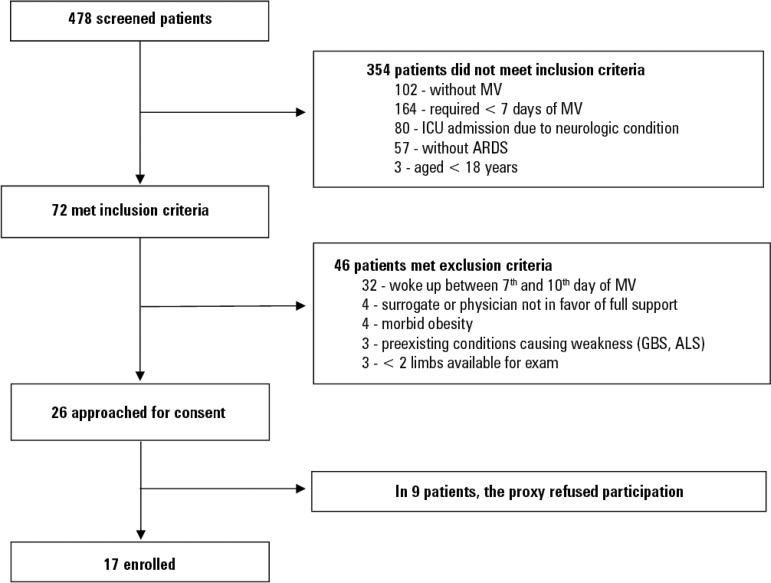
Patients' enrolment. MV - mechanical ventilation; ICU - intensive care unit; ARDS - acute respiratory distress syndrome; GBS - Guillain-Barre Syndrome; ALS - amyotrophic lateral sclerosis.

Seventeen individuals were enrolled during the seven months of the study (Figure 1). The patient characteristics are shown in table 1. Eight patients (47%) were male, the median age was 63.5 ± 16.1 years, and the median APACHE-II score was 21.9 ± 5.7. At the time of electromyographical assessment (between the 7^th^ and the 10^th^ day of MV), 9 patients (53%) were still using vasopressors, and 10 patients (58%) needed dialysis.

**Table 1:** Baseline characteristics of patients

Number of patients	Sex	Age	Site of sepsis	APACHE II	Previous diseases	At 7^th^ MV-day			ICU-outcome	Electrophysiological findings of peripheral muscles
						SOFA	Using norepinephrine	Dialysis		
1	Female	76	Pulmonary	22	CAD	7	Yes	Yes	Dead	CIM
2	Female	66	Pulmonary	18	Asthma	6	Yes	Yes	Dead	CIP + CIM
3	Female	73	Pulmonary	19	No	5	Yes	Yes	Dead	CIP
4	Male	41	Renal	11	No	7	No	Yes	Alive	CIM
5	Female	33	Abdominal	21	No	5	Yes	No	Dead	CIP + CIM
6	Female	41	Abdominal	20	No	6	Yes	No	Alive	Normal
7	Female	72	Pulmonary	25	COPD	7	No	No	Dead	CIM
8	Female	63	Renal	30	CAD	7	Yes	No	Dead	CIP
9	Female	64	Pulmonary	19	COPD	7	Yes	No	Alive	Normal
10	Male	73	Abdominal	20	COPD	7	Yes	Yes	Dead	CIP
11	Male	57	Abdominal	18	No	5	No	No	Alive	Normal
12	Male	66	Renal	22	Alcoholism, COPD	4	Yes	Yes	Alive	Normal
13	Female	76	Abdominal	22	COPD	7	No	Yes	Dead	Normal
14	Male	70	Cerebral	17	Alcoholism, CAD	6	No	No	Alive	CIP + CIM
15	Male	65	Renal	20	No	6	Yes	Yes	Alive	Normal
16	Male	62	Pulmonary	22	CAD	5	No	No	Dead	Normal
17	Male	52	Abdominal	17	No	5	Yes	Yes	Alive	Normal

APACHE II - Acute Physiologic and Chronic Health Evaluation; MV - mechanical ventilation; SOFA - Sequential Organ Failure Assessment; ICU - intensive care unit; CAD - coronary arterial disease; CIM - critical illness myopathy; CIP - critical illness polyneuropathy; COPD - chronic obstructive pulmonary disease.

**Figure 2:**
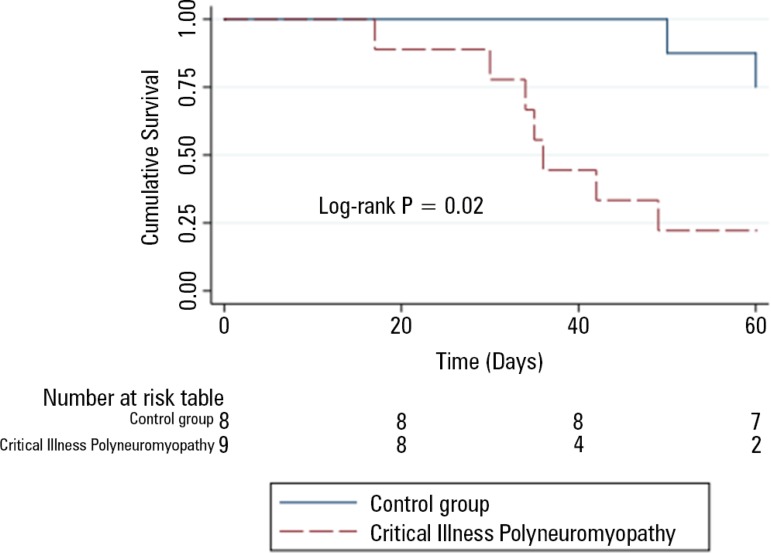
Mortality effect using Kaplan-Meier curves.

Electromyographic signs of CIM or CIP occurred in 9 patients (53%). The overall ICU mortality rate was 53% (9 patients). The ICU mortality was higher in patients with electromyographic diagnoses of CIM or CIP than in those with normal electromyographic studies (77.7% *versus* 12.5%, respectively, log-rank p = 0.02) (Figure 2). Table 2 compares the severity data by ICU outcome.

**Table 2:** Comparison of data by intensive care unit outcome

Characteristics	Alive	Death	p value
Age	55.7 ± 13.8	57.0 ± 15.2	NS
APACHE II	18.1 ± 5.3	22.1 ± 7.6	NS
SOFA score at 7^th^ day	5.7 ± 2.5	6.2 ± 2.7	NS
Norepinephrine use at 7^th^ day	5 (62.5)	6 (66.7)	NS
Dialysis use at 7^th^ day	4 (50.0)	5 (55.5)	NS

APACHE II - acute physiologic and chronic health evaluation; NS - not significant; SOFA - Sequential Organ Failure Assessment. Results expressed as mean ± standard deviation or n (%).

## DISCUSSION

Our findings suggested that electromyographical signs of CIM or neuropathy between days 7 and 10 of MV may be associated with mortality in moderate-severe ARDS (PaO_2_/FiO_2_ < 200) patients under profound sedation.

The occurrence of ICUAW varies substantially depending on the diagnostic method used, the timing of examination, and the type of population under study.^(4,16-18)^ Notably, the focus solely on a clinical-based diagnosis may deprive a high proportion of patients, in whom the clinical strength assessment is not feasible (i.e., patients requiring profound sedation due to unstable ventilator, hemodynamic, or neurologic conditions, or patients unable to collaborate due to acute brain dysfunction or previous cognitive impairment), of an appropriate indicator of worse outcomes. In addition, sedation is often used in the care of mechanically ventilated patients, and there is increasing recognition that the management of such nonventilator aspects of care influences outcomes. A recent meta-analysis^(19)^ showed a significant relationship between early sedation depth and clinical outcomes. Early light sedation was associated with decreased hospital mortality, MV duration, and ICU length of stay compared with early deep sedation. With regards to the patients in the present study, deep sedation may be necessary in moderate-severe ARDS. These patients are at risk of ICUAW, and a method to evaluate ICUAW is of paramount importance. Interestingly, the use of electromyographical findings of CIM or CIP in this subset of patients, in whom the clinical diagnosis of ICUAW is not possible, might improve the early implementation of strategies aimed at mitigating complications related to muscular dysfunction, such as early mobilization, personalized weaning from MV, minimization of exposure to corticosteroids and neuromuscular blockers, and early tracheostomy.^(20-22)^ Future studies may focus on the lack of personalized rehabilitation strategies guided by electromyographical assessment of relevant outcomes, such as length of MV and ICU stay.

Our findings regarding the association between CIM and CIP and higher mortality are consistent with previous studies. Khan et al.^(23)^ carried out electromyographical studies on 48 patients with severe sepsis and found that abnormal nerve conduction within 72 hours of ICU admission predicted hospital mortality (55% *versus* 0%, p < 0.001). The outcome of these patients was associated with a prolonged duration of MV and increased hospital and ICU stays. The study by Garnacho-Montero et al.^(24)^ on 64 critically ill septic patients showed that the duration of the weaning period was significantly higher in patients with CIM or CIP than those without (median 15 days *versus* 2 days, respectively, p < 0.001). Multiple logistic regression analysis indicated that CIP was the only risk factor independently associated with weaning failure (odds ratio, 15.4; 95% confidence interval -95%CI, 4.55 - 52.3, p < 0.001). In our study, the time frame of 7 to 10 days for CIM and CIP diagnosis was chosen based on previous studies. Latronico et al.,^(25)^ for example, in a prospective and multicenter study, conducted electromyographical studies on a daily basis on patients admitted to the ICU, and showed that electrophysiological signs of CIM or CIP were present in 30.4% (95%CI 21.9% - 40.4%) of patients, with a median time from ICU admission to CIM or CIP of six days (95%CI, 5.0 - 9.0).

The strengths of our study are as follows:

- Evaluation of moderate-severe ARDS patients. The pathophysiologic mechanisms leading to weaning failure and a high rate of mortality in this group may be complex and depend on many factors, with several potentially reversible etiologies for weaning failure, including respiratory and/or cardiac load; neuromuscular competence (central and peripheral); critical illness neuromuscular abnormalities; neuropsychological factors; and metabolic and endocrine disorders. In addition, weaning dependency from MV is a landmark of chronic critical illness, an independent predictor of mortality.^(26)^
- Inclusion of mechanically ventilated patients with profound sedation. Neurological examination is often unreliable in mechanically ventilated patients due to encephalopathy, sedation, or the critical condition of the subject; therefore, comprehensive electrophysiological studies of peripheral nerves may be necessary to establish the diagnosis and prognosis of critical illness patients in this context.^(17)^

Our study also has significant limitations: the small number of patients limited the use of covariate adjustment and, therefore, might have resulted in an inaccurate risk measure. Additionally, the small sample size limits the generalization of our study findings; absence of data on important covariates such as muscle biopsy, MV variables and biomarkers; no assessment of the muscle contraction sequence to compare spontaneous and supported ventilation in awake patients; and absence of other important outcomes that could reinforce the link between CIM and CIP and mortality.

## CONCLUSION

The electromyographical signs of critical illness myopathy or neuropathy may be associated with intensive care unit mortality among moderate-severe acute respiratory distress syndrome patients.
